# Perception of stress-related working conditions in hospitals (iCept-study): a comparison between physicians and medical students

**DOI:** 10.1186/1745-6673-8-3

**Published:** 2013-02-26

**Authors:** Jan Bauer, David A Groneberg

**Affiliations:** 1 Institute of Occupational, Social and Environmental Medicine, Goethe-University, Theodor-Stern-Kai 7, 60329, Frankfurt am Main, Germany

**Keywords:** Perception, Stress, Working conditions, Hospital, Student

## Abstract

**Background:**

The students’ perception of working conditions in hospitals hasn’t been subject of research in Germany so far. However the perception plays an important role talking about the sustainability of working conditions. The iCept Study wants to examine the perception of medical students compared to the perception of practicing physicians.

**Methods/design:**

The perception will be investigated with a redesigned questionnaire based upon two established and validated questionnaires. The two samples built for this study (students and physician) will be chosen from members of the labor union Marburger Bund. The iCept-Study is designed as an anonymized online-survey.

**Discussion:**

The iCept-Study is thought to be the basis of ongoing further investigations regarding the perception of working conditions in hospitals. The results shall serve the facilitation of improving working conditions.

## Background

There are three crucial aspects concerning working conditions in the context of their sustainability and their influence on medical students: First, the physicians, who set the students an example of current working conditions. Second, the students corresponding perception of the working conditions. Third, the resulting needs and expectations of medical students about their future working conditions. The first and the latter aspect has been subject of many research studies [1-5]. However, the second one as a link between the current and to-be analysis, hasn’t been a subject of scientific research in Germany so far. The iCept-Study wants to examine the students’ perception of working conditions, answering the following pivotal questions:

1. How do medical students perceive stress-related working conditions of their supervising physicians?

2. Is the perception realistic?

3. Are there differences in the perception regarding age, specialty or state?

Generally speaking, the perception of working conditions depends on the direct observation of physicians at work and on the informal information from the peer group or the media who is influencing the perception of medical students [6-9].

Whether the perception is congruent with reality cannot be answered with current data. Knowing that the perception has a major impact on the specialty choice of medical students makes the importance even more evident [6]. Furthermore there is an upcoming shortage of qualified employees in German hospitals: The “Deutsches Krankenhausinstitut” (DKI) assumes a further requirement of 37.370 physicians until 2019 [10]. In cooperation with PricewaterhouseCoopers the institute for economic research (WifOR) estimated an additional need of 56.000 physicians until 2020 and 106.000 until 2030 [11,12]. The “Kassenärztliche Bundesvereinigung” and the “Bundesärztekammer” quantified the need to an extend to 71.625 missing physician until 2020 [13]. These data suggest a threatening shortage of qualified medical employees and thus an urgent need for motivated medical students, willing to work in hospitals.

In this study, two stress models were used for determining stress-related working conditions.

1. The job-demand-control (JDC) model of Karasek et al.: In this theoretic model two parameters are confronted. On the one side the “job demand” and on the other side the “control” in terms of scope of action respectively scope for decision-making. Karasek et al. postulate that an imbalance between too high “job demand” and too little “control” (JDC-ratio > 1) results in “mental strain” [14,15]. A current survey from 2012 interviewed medical employees in hospitals and proved the importance of the JDC model regarding the development of stress-related symptoms [16].

2. The effort-reward-imbalance (ERI) model of Siegrist et al.: This model postulates an imbalance between the “effort” at work and the corresponding “reward” as an intrinsic stress factor with all its negative psychological and physical manifestations. The negative consequences develop from domination of the “effort” in relation to the “reward” (ER-ratio > 1). There are three different types of “reward”: money, respect/acknowledgment and career advancement [17]. A 2006 published meta-analysis showed that high job demands, lack of social support, job insecurity and low appreciation raised the incidence rate of mental illnesses [18].

In the iCept-Study both models are combined, since thereby both extrinsic (JDC) and intrinsic (ERI) stress factors are taken into account. The importance of both models on the well being of employees was shown in a study that examined their influence on the incident rate of myocardial infarction [19].

## Methods

The iCept-Study is designed as an anonymized online-survey. Therefore the study is orientated towards the “international codex of market and social research” and, because it will be administered in Germany, towards the respective declaration for the federal republic of Germany [20,21]. Furthermore the “standards for quality assurance of online-surveys” will be taken into account [22]. The necessary scientific standards of quality can be found in the “Norm DIN ISO 20252:2006; Markt-, Meinungs- und Sozialforschung – Vokabular und Anforderungen”.

### Sample

In this Study two samples will be recruited: physicians and medical students. The sample of physicians will be used as the control-group, the medical students as the experimental-group. Both samples will be chosen randomly from the members of the Marburger Bund, a professional organization and labor union of employed physicians. On the cut-off date, the July 1^st^ 2012, the Marburger Bund had 83.123 physicians and 19.223 medical students as members. The members will be contacted through e-mail in a standardized form, which will be distributed with the kind support of the Marburger Bund. The e-mail will contain a personalized link to the iCept-Study. In addition, Marburger Bund internal media like the “MB-Newsletter” or the “Marburger Bund Zeitung (MBZ)” will be used. This inclusion criterion is out of date and will not be used in this study, since medical students DO have relevant clinical internships in the younger semesters.

### ICept questionnaire

The iCept Questionnaire is built to assess mainly the above-mentioned two theoretic stress models (JDC and ERI). For that purpose the questionnaire is based upon the short questionnaire for work place analysis (KFZA) of Prümper et al. [23] and the questionnaire for the effort-reward-imbalance (ERI) of Siegrist et al. [24].

The KFZA is an established and validated questionnaire since 1995 and has been used in many studies especially in hospitals [25-27]. Moreover it is listed by the federal institute of work safety and occupational medicine (BAuA) as a universal screening method with satisfying quality criteria [28]. The KFZA is also the basis for the “IMPULS-Test” of Molnar et al. and other questionnaires [29-31]. It consists of 26 items and 11 scales.

The effort-reward-imbalance questionnaire (ERI-questionnaire) by Siegrist et al. has been developed in 2004 to assess the identical stressor (ERI). The quality criteria are satisfying (Crohnbach’s α > 0,7). The answer format is a 5-point Likert scale, whereas current data suggest a 4-point Likert scale to be more suitable [24,32]. The questionnaire exists in a long (26 items) and a short (16 items) version [32,33]. The short version has been used in many different studies [34,35] and is also listed by the federal institute of work safety and occupational medicine (BAuA) as a screening method with satisfying quality criteria [36].

Developing the iCept questionnaire, the items of KFZA and ERI-questionnaire have been reviewed for practicability at the clinical workplace. Also the items must be answerable for medical students from their point of view. Only items and scales fulfilling these criteria were used. The kind approval from Prof. Jochen Siegrist (ERI-questionnaire) and Prof. Andrea Abele-Brehm (adapted KFZA) to use their questionnaires in this study has been obtained.

The stressors defined by the JDC are covered by the KFZA: the “job demand” is measured through the scales “suitably demanding work” (QL1/QL2) and “suitable volume of work” (QN1/QN2); the “control” is assessed through the scale “scope for action” (HS4-HS6) [37]. In addition, the scales “cooperation” (ZU2/ZU3) and “social support” (SR1-SR3) are taken from the KFZA. The items HS4-HS6 and an additional scale, “social climate” (SK1, SK2), have been taken from the adapted version of KFZA by Abele [31].

There is a slight correlation of both, ERI and KFZA questionnaires, regarding the scales “job demands” and “effort”. Different studies showed a correlation between the scales from r=0,3 to r=0,6 [38,39]. Thus the scale “effort” is measured by items of both questionnaires (ERI2/ERI5 and QN1/QN2). Also the scale “reward” is measured by both questionnaires (ERI7/ERI8/ERI10 and SR1/SR2).

The overall job satisfaction is measured by a single item (JS1) from the “Job Diagnostic Survey” (JDS) of Schmidt et al. [40] That a single item can be used to measure the job satisfaction has been shown in a meta-analysis, postulating a correlation of r=0,67 between “single-item measures” and “scale measures” regarding job satisfaction [41].

The sociodemographic data are assessed according to the “demographic standards” of the federal institution of statistics [42]. The following data will be collected:

•EM1: Gender

•EM2: Age

•EM3: Specialty

•EM4: State

•EM5: Semester (only students)

•EM6: Position (only physician)

**Figure 1:**
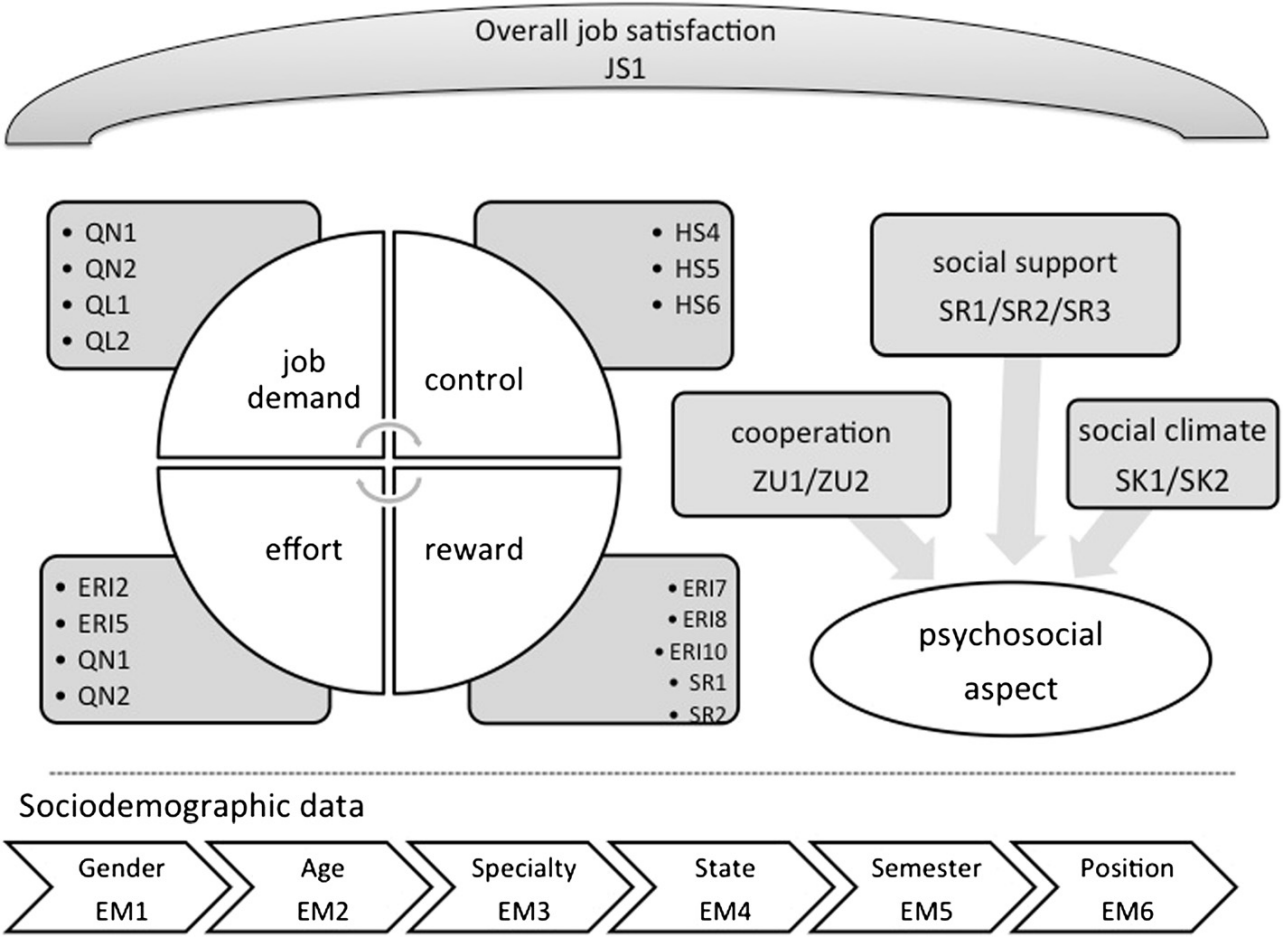
Allocation of iCept-items to target parameter.

Figure 1 shows all items with their target parameter.

There will be two slightly different questionnaires administered: one for medical students and one for physicians. The items of both versions only differ grammatically but not content wise or semantically: The items for the physicians’ version will be written in the first-person singular, whereas the version for students will be in the third-person singular. So there is no change to any substantial degree. The items will be answered on a 4-point Likert scale (strongly disagree, disagree, agree, strongly agree). The complete iCept questionnaire contains 20 items and 5 more sociodemographic items (see Table  1) and will take about 5–10 min of the participants’ time.

**Table 1:** iCept-questionnaire for medical students

**Item code**	**Item**
HS4	The physician can determine the sequence of his work steps.
HS5	The physician has much influence as to which task is allocated to him.
HS6	The physician is free to plan and classify his task independently.
SR1	The physician can rely on his colleagues when work becomes difficult.
SR2	The physician can rely on his immediate superior when work becomes difficult.
SR3	The team spirit is good in the department.
ZU2	The physician can talk to other colleagues during work about official and personal matters.
ZU3	The physician always gets a feedback from superiors and colleagues on the quality of his work.
QL1	The work involves things that are too complicated
QL2	The demands made on the physicians’ concentration are too high.
QN1	The physician is often under time pressure
QN2	The physician has too much work
SK1	At the physicians’ workplace, there is a strong competition.
SK2	The social climate at the physicians’ workplace is burdensome.
ERI2	The physician has many interruptions and disturbances while performing his job.
ERI5	The physicians’ job is physically demanding.
ERI7	The physician receives the respect he deserves from his superiors.
ERI8	The physician receives the respect he deserves from his colleagues.
ERI10	The physician is treated unfairly at work.
JS1	Generally speaking, the physician is very satisfied with his job.
	Sociodemographic data
EM1	Gender
EM2	Age
EM3	Specialty
EM4	State
EM5	Semester

In order to keep the influence of the peer group or the media on the students’ perception as low as possible, the students will be asked only to rate the latest clinical internship.

The survey will be generated with the web based online survey tool “2ask” from the amundis communications GmbH. The Leibniz institute for social science recommends this tool [43].

### Statistical data analysis

The statistical data analysis will be performed with SPSS Statistics. As Figure 1 shows, the scales “effort” and “job demand” are measured by 4 items, the scale “reward” by 5 items and the scale “control” by 3 items. Considering the 4-point Likert scale, the scale sum scores varies:

• Scale sum score “effort” (x_eff_):4 ≤ x_eff_ ≥ 16

• Scale sum score “job demand” (x_job_):4 ≤ x_job_ ≥ 16

• Scale sum score “reward” (x_rew_):5 ≤ x_rew_ ≥ 20

• Scale sum score “control” (x_con_):3 ≤ x_con_ ≥ 12

In order to draw first conclusions about the stressors ERI and JDC the ratio between the respective scale sum scores are calculated (ER-ratio and JDC-ratio). To adjust the unequal number of items a correction factor, based on the number of items, is used (c_eri_=1,25 for the scale “effort” and c_jdc_= 0,75 for the scale “job demand”).

$$\begin{array}{cc} \text{ER}-\text{Ratio}=\frac{x_{\text{eff}}}{x_{\text{rew}}}\times c_{\text{eri}} & \text{JDC}-\text{Ratio}=\frac{x_{\text{job}}}{x_{\text{con}}} \end{array}\times c_{\text{jdc}}$$

Values > 1 of the ER/JDC-ratio indicate stress with possible adverse health effects [24,44].

Besides this relative component, indicating an imbalance between the scales, the absolute component will also be calculated, indicating possible eustress. For this purpose the sum scale scores of “effort” and “reward” respectively “job demand” and “control” will be summed up (ER-Sum, JDC-Sum).

$$\begin{array}{ll} \text{ER}-\text{Sum}=\frac{x_{\text{eff}}}{4}+\frac{x_{\text{rew}}}{5} & \text{JDC}-\text{Sum}=\frac{x_{\text{job}}}{4}+\frac{x_{\text{con}}}{3} \end{array}.$$

For the analysis values > 5 (as a sufficient condition) and an ER/JDC-ratio = 1 (as a necessary condition) will be taken as an indicator for healthy stress (eustress).

Seven items (ZU1/ZU2, SK1/SK2, SR1-SR3) reflect the psychosocial aspect of stress and will be analyzed separately as well as the “overall job satisfaction” item (JS1).

## Discussion

The iCept-Study is thought to be the basis of ongoing further investigations regarding the perception of working conditions in hospitals. The results shall serve the facilitation of improving working conditions. Especially the rough transition from medical school to the first job in a hospital makes it very important to know the students’ perception in order to smooth that transition. Another implication the perception of working conditions has, is the influence on the students’ specialty choice. This hasn’t been a subject of research in Germany so far and therefor could be considered as a future subject in the iCept-Study.

## Abbreviations

DKI: Deutsches krankenhausinstitut; WifOR: Wirtschaftsforschungsinstitut; JDC: Job-demand-control; ERI: Effort-reward-imbalance; MB: Marburger bund; MBZ: Marburger bund zeitung; KFZA: Kurz-fragebogen zur arbeitsanalyse; BAuA: Bundesanstalt für arbeitschutz und arbeitsmedizin.

## Competing interests

The author declares that he has no competing interests.

## Authors’ contributions

JB conceived and designed the study and wrote the manuscript. DAG contributed to its final version. All authors read and approved the final manuscript.
